# Atrial fibrillation and microRNAs

**DOI:** 10.3389/fphys.2014.00015

**Published:** 2014-01-24

**Authors:** Gaetano Santulli, Guido Iaccarino, Nicola De Luca, Bruno Trimarco, Gianluigi Condorelli

**Affiliations:** ^1^Department of Advanced Biomedical Sciences, “Federico II” University HospitalNaples, Italy; ^2^Department of Translational Medical Sciences, “Federico II” University HospitalNaples, Italy; ^3^Columbia University Medical Center, College of Physicians & Surgeons, New York Presbyterian Hospital - ManhattanNew York, NY, USA; ^4^Department of Medicine and Surgery, University of SalernoSalerno, Italy; ^5^IRCCS “Multimedica,”Milano, Italy; ^6^Humanitas Clinical and Research CenterRozzano (Milan), Italy; ^7^University of MilanMilan, Italy

**Keywords:** atrial fibrillation, microRNA (miRNA), electrical remodeling, apoptosis, structural remodeling, electrophysiology, fibrosis

## Abstract

Atrial fibrillation (AF) is the most common sustained arrhythmia, especially in the elderly, and has a significant genetic component. Recently, several independent investigators have demonstrated a functional role for small non-coding RNAs (microRNAs) in the pathophysiology of this cardiac arrhythmia. This report represents a systematic and updated appraisal of the main studies that established a mechanistic association between specific microRNAs and AF, focusing both on the regulation of electrical and structural remodeling of cardiac tissue.

## MicroRNA

MicroRNAs (miRs) are an evolutionarily conserved class of small (~22 nucleotides) non-coding RNAs (Ambros, 2004; Gan et al., 2013), first discovered in *Caenorhabditis elegans* (Ruvkun and Giusto, 1989; Ruvkun et al., 1989). They represent a vital component of genetic regulation, existing in virtually all organisms, suggesting thereby a pivotal role in biological processes (Latronico and Condorelli, 2008; Thum et al., 2008). Indeed, miRs are important regulators of gene expression in numerous biological processes including cellular proliferation, differentiation, and tumorigenesis (Care et al., 2007; Dvinge et al., 2013; Shen et al., 2013; Song et al., 2013). Typically, miRs are regarded as negative regulators of gene expression that inhibit translation and/or promote mRNA degradation by base pairing to complementary sequences within the 3′-untranslated region (3′-UTR) of protein-coding mRNA transcripts (Van Rooij and Olson, 2012; Meijer et al., 2013). Generally, mRNA degradation accounts for the majority of miR activity (Guo et al., 2010). Hence, by altering levels of key regulators within complex genetic pathways, miRs provide a posttranscriptional level of control of homeostatic and developmental events (Callis et al., 2009; Yates et al., 2013).

It is estimated that miRs regulate over 60% of all protein-coding genes (Friedman et al., 2009; Akerman and Mukherjee, 2013; Leucci et al., 2013). Considering that a single miR can regulate multiple mRNAs and that each mRNA may be a target of multiple miRs, the possible pathways for miR-dependent regulation of protein abundance seem to be extremely complicated (Akerman and Mukherjee, 2013; Santulli and Totary-Jain, 2013). In this model, a biologic response would be expected only after co-expression of various miRs that cooperatively target different components of a functional network (Liu et al., 2012; Van Rooij and Olson, 2012) or are all required to sufficiently repress a single target (Lagos-Quintana et al., 2001; Kim, 2013).

## Biogenesis and biological action of miRs

Maturation of miRs involves a multi-stepped process (Bartel, 2004; Cullen, 2004) that starts from the transcription (mainly operated by RNA polymerase II) of single-stranded non-protein-coding RNAs, which are either transcribed as stand alone transcripts (*intergenic* miRs), often encoding various miRs, or generated by the processing of introns of protein-coding genes (*intragenic* or intronic miRs).

Transcription of intergenic miRs leads to the formation of primary miRs (pri-miR) with a characteristic hairpin or stem-loop structure (Denli et al., 2004), which are subsequently processed by the nuclear RNase III, Drosha (Zeng et al., 2005), and its partner proteins, among which there is the DiGeorge Syndrome Critical Region 8 (DGCR8, known as *Pasha* in invertebrates), named for its association with DiGeorge Syndrome (Shiohama et al., 2003; Roth et al., 2013), to become precursor miR s (pre-miR). On the other hand, intronic miRs are obtained by the regular transcription of their host genes and then spliced to form looped pre-miRs, bypassing thereby the Drosha pathway (Bartel, 2004; Ruby et al., 2007).

Pre-miRs are exported from the nucleus in the cytoplasm in a process involving the Ran-GTP-dependent shuttle Exportin-5 (Lund et al., 2004). Once in the cytosol, the pre-miR hairpin is cleaved by the RNase III enzyme Dicer (Saxena and Tabin, 2010; Marasovic et al., 2013), yielding a mature miR:miR^*^ duplex about 22 nucleotides in length, which is subsequently incorporated into the protein complex called RNA-induced silencing complex (RISC) to form miRISC (Filipowicz et al., 2008; Wu et al., 2013). At this point, one of the double strands, the guide strand, is selected by the argonaute protein (Pfaff et al., 2013), the catalytically active RNase in the RISC complex, on the basis of the thermodynamic stability of the 5′ end. In particular, the strand with a less thermodynamically stable 5′ end is commonly chosen and loaded into the RISC complex (Siomi and Siomi, 2009), serving as a guide for mRISC to find its complementary motifs in the 3′-UTR of the target mRNA(s). Although either strand of the mature duplex may potentially act as a functional miR, only one strand is usually incorporated into the RISC where the miR and its mRNA target interact (Fabian and Sonenberg, 2012; Von Brandenstein et al., 2012). Such a binding inhibits the translation of the protein that the target mRNA encodes or promotes gene silencing via mRNA degradation (Latronico and Condorelli, 2008; Kallen et al., 2012; Papait et al., 2013).

In the human genome more than 1200 miR sequences have been identified, hitherto [miRTarBase, Release 4.5, version 20 (Hsu et al., 2011)], with over 50,000 miR-target interactions. Recently, several algorithms and bioinformatics websites, including TargetScan and miRWalk (Lewis et al., 2005; Dweep et al., 2011) have been developed to predict specific mRNA/miR interactions. However, miR-binding rules are quite complex and are not fully understood, resulting in a lack of consensus in the literature.

Given all these crucial features, miRs could represent an important way for the cell to establish intercellular (with other cells, via secreted miRs) and intracellular (among its own genes) communication.

Establishing direct cause-and-effect links between miRs and mRNA targets is essential to understanding the molecular mechanisms underlying disease and the subsequent development of targeted therapies (Ambros et al., 2003; Zacharewicz et al., 2013).

## Atrial fibrillation

Atrial fibrillation (AF) is a highly prevalent disease with a significant genetic component (Den Hoed et al., 2013; Mahida, 2013; Santulli, 2013), considered the most common sustained arrhythmia, which can cause or exacerbate heart failure and represents an important risk factor for ischemic stroke (Fye, 2006; Conen et al., 2011; Santulli, 2012b; Santulli et al., 2013; Thomas and Sorrentino, 2014). AF represents the most commonly seen arrhythmia worldwide, especially in the geriatric population (Huikuri, 2008; Riley and Manning, 2011; Santulli et al., 2012c; Santulli and Iaccarino, 2013) and is associated with a substantially pronounced morbidity and mortality (Beyerbach and Zipes, 2004; Santulli, 2012a; Garg and Akoum, 2013; Menezes et al., 2013). From a pathophysiological point of view, AF is characterized by atrial electrical remodeling, mainly mediated by ion-channel alterations (Brundel et al., 2001; Santulli et al., 2012b; Xie et al., 2013) and structural remodeling (fibrosis and apoptosis), which favors arrhythmia recurrence and maintenance (Perino et al., 2011; Santulli and D'Ascia, 2012; Santulli et al., 2012b). A noticeable feature of the electrical remodeling associated with AF is the abbreviation of the effective refractory period favoring reentry (D'Ascia et al., 2010, 2011; Kapur and Macrae, 2013), primarily due to shortening of atrial action potential duration (APD).

Three potential models have been proposed to explain the pathophysiology of AF (Jalife, 2011), albeit the precise relationship of each of these conceptual frameworks to human AF remains under investigation (Vikman et al., 2005; Kapur and Macrae, 2013; Shah et al., 2013):

The focal mechanism theory suggests that AF is provoked by the rapid firing of single or multiple ectopic foci, and also proposes a functional role for continued ectopic firing in the maintenance of AF (Lee et al., 2013).

The single circuit re-entry theory of AF assumes the presence of a single dominant reentry circuit alongside with the fragmentation of emanating waves in the heterogeneous electrical substrate of normal atrial tissue (Zemlin and Pertsov, 2007; Kapur and Macrae, 2013).

The multiple wavelet theory of AF stands on the notion that multiple reentry circuits exist, with randomly propagating wavefronts that must find receptive tissue in order to persist (Haissaguerre et al., 2013).

Slowing of conduction velocities and shortening of the refractory period of atrial myocytes (both central features of the electrical remodeling seen in AF) might help to stabilize the arrhythmia by decreasing circuit size. Of course these mechanistic models are not mutually exclusive. They may coexist in a single subject at various stages in the pathogenesis of AF and each may be applicable to certain subgroups of AF patients (Lindgren et al., 2003; Brieger and Freedman, 2009; Ruwald et al., 2013). Theoretically, all the miRs that are directly or indirectly involved in one of these processes, which are eventually based on the regulation of structural or electrical remodeling (cardiac automaticity, ion channels, fibrosis, and apoptosis), could participate in AF induction or perpetuation.

## Experimental strategy to identify miRs involved in human disease

The most common experimental approach to identify the specific miRs that play a role in a certain disease mainly consists of three phases: (1) Use a microarray matrix to recognize a list of miRs that are differentially expressed in subjects with the disease compared to control subjects (Frezzetti et al., 2011; Jayaswal et al., 2011); (2) assess the putative target site efficacy by using bioinformatics-based algorithms or other computational tools that score potential interactions between microRNAs and mRNAs (Witkos et al., 2011); (3) validate *in vitro* (or *in vivo*) the existence of an inverse correlation between the expression levels of the miR and protein levels of its target gene(s). Another biological validation could be also achieved using a reporter system or other assays to prove that the binding of the miR and the target mRNA occurs within a RISC complex (Ling et al., 2013).

## Functional role of miRs in atrial fibrillation

Growing evidence demonstrates that miRs regulate several properties of cardiac physiology and excitability, including automaticity, Ca^2+^ handling, conduction, and repolarization (Grueter et al., 2012; Boon et al., 2013; Heymans et al., 2013; Latronico and Condorelli, 2013). In particular, recent reports have unveiled an essential role of miRs in regulating cardiac excitability and arrhythmogenesis (Callis et al., 2009; Shan et al., 2009). These studies have primarily focused on the two muscle-specific miRs, i.e., miR-1 and miR-133, which are among the most abundantly expressed miRs in the heart (Liang et al., 2007; Wang et al., 2011).

However, lately other ubiquitously distributed miRs, such as miR-328 have been shown to exhibit a strong arrhythmogenic potential (Lu et al., 2010). It is likely that multiple miRs contribute to controlling arrhythmogenicity of the heart and that different miRs are involved in different types of arrhythmias under different pathological conditions of the heart (Horie et al., 2012; Kochegarov et al., 2013; Qiao et al., 2013; Zhang et al., 2013a). The most important miRs so far implicated in the pathophysiology of AF, regulating both electrical and structural remodeling, are reported in Table 1, alongside with their target gene(s) and function(s).

**Table 1:** **miRs with an established role in the regulation of cardiac electrical and structural remodeling**.

**miR**	**Changes in AF**	**Main target genes and their function**		**References**
miR-1	Down-regulated	KCNJ2	Increased I_K1_	Zhao et al., 2007; Girmatsion et al., 2009
		GJA1 (connexin43)	Altered conduction	
		Fibullin-2	Increased fibrosis	
miR-21	Up-regulated	Spry1, PDCD4	Inhibition of fibroblast proliferation	Adam et al., 2012
miR-26	Down-regulated	KCNJ2	Increased I_K1_	Luo et al., 2013
miR-29	Down-regulated	Fibrillin, collagen-1A1, collagen-3A1, Mcl-2	Increased fibrosis	Dawson et al., 2013
miR-30	Down-regulated	CTGF	Increased fibrosis	Duisters et al., 2009
miR-133	Down-regulated	CTGF, TGF-β	Increased fibrosis	Cooley et al., 2012
miR-328	Up-regulated	CACNB1	Shortened atrial action potential duration	Lu et al., 2010
		CACNA1C		
miR-499	Up-regulated	KCNN3	Altered conduction	Ling et al., 2013

*AF, Atrial fibrillation; KCNJ2, K^+^ inwardly-rectifying channel, subfamily J, member 2; GJA1, Gap junction alpha1 protein; SPRY1, sprouty homolog 1; Mcl-2, Myeloid cell-leukemia-2; CTGF, Connective tissue growth factor; TGF-β, Transforming growth factor β ; KCNN3, K^+^ intermediate/small conductance Ca^2+^-activated channel*.

Although circulating miRs seem to be interesting candidates as biomarkers in AF patients, they still possess several important limitations (Quiat and Olson, 2013; Santulli and Totary-Jain, 2013). Indeed, at the moment there is no good natural stable housekeeping control for circulating miRs, which may result in strong variations, and they are generally present in low amounts in plasma and serum.

## Regulation of proteins involved in electrical remodeling by miRs

As mentioned above, arrhythmogenesis is essentially attributed to enhanced triggered activity, and several studies have attributed such activity to alterations in different ion channels, including a peculiar instability in Ca^2+^ handling (Ter Keurs and Boyden, 2007; Zhang et al., 2008; Latronico and Condorelli, 2009; Kushnir and Marks, 2010; Nivala et al., 2012) and modulation of the cardiac inwardly rectifying K^+^ current (I_K1_), which stabilizes the resting membrane potential, controls cardiac excitability, regulates late-phase repolarization, and is thereby responsible for shaping the initial depolarization and final repolarization of the action potential (Dhamoon and Jalife, 2005).

In a seminal study, the expression of miR-1 was demonstrated to be reduced by ~86% in tissue samples of AF patients (Girmatsion et al., 2009). This miR has been shown to be mechanistically critical in the pathophysiology of AF via targeting *I_K1_* and connexin43 (Girmatsion et al., 2009), both considered master regulators of cardiac conduction (Delmar and Makita, 2012; Musa et al., 2013). Similarly, miR-26 has the potential to repress *I_K1_* (Luo et al., 2013). Intriguingly, in the setting of ventricular arrhythmias miR-1 levels appear to be augmented (Anderson and Mohler, 2007; Yang et al., 2007). Thus, the different regulation of such a miR in ventricular and atrial tissue deserves further investigations (Anderson and Mohler, 2007; Yang et al., 2007; Santulli et al., 2012a). The recently proposed involvement of miR-1 in the modulation of Ca^2+^ handling proteins, including calmodulin, phospholamban, Na^+^/Ca^2+^ exchanger (NCX), sorcin, junctin, triadin, eventually resulting in shortened refractoriness of sarcoplasmic reticulum Ca^2+^ release further support the hypothesis of a pivotal functional role of such a miR in the pathogenesis of AF (Ali et al., 2012; Karakikes et al., 2013; Slagsvold et al., 2013; Tritsch et al., 2013; Zhang et al., 2013b).

Lu et al. (2010) demonstrated that miR-328 level was elevated in AF patients and identified as target genes CACNA1C and CACNB1, encoding L-type Ca^2+^ channel subunits. Thereby, increased levels of miR-328 reduce *I*_CaL_ density and shorten APD, leading to an increased arrhythmogenic potential. Additionally, miR-223, miR-328, and miR-664 were found to be upregulated by >2 fold in AF samples (Lu et al., 2010), and further investigations are required to establish the molecular mechanism underlying such a change in the miR transcriptome.

Most recently, Ling and colleagues found a strong association between mir-499, which is significantly up-regulated in atrial tissue from AF patients, and KCNN3, the gene that encodes the small-conductance Ca^2+^-activated K^+^ channel 3 (SK3), possibly contributing to the electrical remodeling observed in AF (Ling et al., 2013)

## Regulation of proteins involved in structural remodeling by miRs

Fibrosis is the hallmark of structural cardiac remodeling (Allessie et al., 2002; Nguyen et al., 2013). Structural changes in the atria of AF patients have been identified (Anyukhovsky et al., 2005) at the level of cardiomyocytes and extracellular matrix (ECM), which predominately includes collagen types I and III, fibronectin, laminin, fibromodulin, and entactin (Goudis et al., 2012). ECM remodeling represents a maladaptive response to changes in myocardial structure and function during the progression of cardiac disease (Siwik and Colucci, 2004; Dernellis and Panaretou, 2006). Degradation and deposition of ECM is a process dynamically regulated by a delicate balance (Goudis et al., 2012) between matrix metalloproteinases (MMPs) and tissue inhibitors of matrix metalloproteinases (TIMPs).

In addition to the regulation of key proteins involved in electrical remodeling, miR-1 also modulate cardiac fibrosis, through means of its target protein Fibullin-2, a secreted protein implicated in ECM remodeling (Karakikes et al., 2013). An intriguing role for miR-21 and its downstream target Sprouty (Spry1), a master regulator of fibroblast survival and growth factor secretion, controlling the extent of interstitial fibrosis (Thum et al., 2008), has been demonstrated both in AF patients, in which atria miR-21 is increased, and murine models (Adam et al., 2012). This miR might also be involved in the apoptosis process and in inflammation (Ando et al., 2013). Several groups have recently proposed miR-29 as a mechanistic contributor in AF, through means a regulation of several proteins involved in cardiac fibrosis and apoptosis (Straten and Andersen, 2010; Dawson et al., 2013; Hale and Levis, 2013).

Another potential therapeutic target to modulate fibrosis in AF is miR-30, which expression is down-regulated in AF. This miR directly interacts with the 3′ untranslated region of the connective tissue growth factor (CTGF), a key profibrotic protein (Duisters et al., 2009). The same group also proposed miR-133 as a modulator of CTGF protein levels (Duisters et al., 2009). Of interest, the same miR-133, which levels are reduced in AF patients (Cooley et al., 2012), is involved in the regulation of apoptosis and TGF-β signaling (Goette, 2009).

## Conclusive remarks

Mounting evidence demonstrates that miRs are becoming one of the most fascinating areas of biology, given their critical roles in fine-tuning of physiological processes and their deregulation in several disorders, including AF. The relative role of different miRs in AF may also depend on the underlying etiology of AF, as the rhythm is an end stage manifestation of multiple, distinct predisposing pathological changes. The functional role of miRs as direct or indirect post-transcriptional regulators of genes implied in electrical and/or structural remodeling strongly suggest that these miRs may serve as potential biomarkers or promising drug targets, in prevention, treatment, and management of AF.

### Conflict of interest statement

The authors declare that the research was conducted in the absence of any commercial or financial relationships that could be construed as a potential conflict of interest.
